# Effect of maternal active smoking during pregnancy on the trajectory of childhood body mass index: A multilevel analysis using quartiles of birthweight

**DOI:** 10.18332/tid/119117

**Published:** 2020-04-24

**Authors:** Miho Kamiya, Kohta Suzuki, Zentaro Yamagata

**Affiliations:** 1 Department of Health and Psychosocial Medicine, Aichi Medical University School of Medicine, Nagakute, Japan; 2 Department of Child and Family Health Nursing, Faculty of Nursing, Japanese Red Cross College of Nursing, Shibuya, Japan; 3 Department of Health Sciences, Graduate School of Medicine, University of Yamanashi, Chuo, Japan

**Keywords:** birthweight, pregnancy, active smoking, childhood growth, body mass index

## Abstract

**INTRODUCTION:**

Maternal active smoking during pregnancy is associated with childhood obesity; however, whether maternal active smoking affects childhood body mass index (BMI) according to birthweight has not been examined.

**METHODS:**

The study participants were 1955 women and their single-born infants, born between 1 April 1991 and 31 March 2003, in Koshu City, Japan, for whom complete data for birthweight, pre-pregnancy maternal BMI and pregnancy smoking status were available. Maternal smoking status during pregnancy was recorded using a questionnaire at the time of pregnancy registration. Childhood BMI was estimated by the BMI z-score, established by the World Health Organisation. Birthweight quartiles were grouped by sex and parity (first vs second or higher). Multilevel analysis, including both the individual and time as different level variables by each birthweight quartile, was used to describe the trajectories of BMI z-scores for statistical analyses.

**RESULTS:**

In every quartile group, although children born to smoking mothers were leaner at birth, their BMI z-score increased around the age of 3 years. These children were larger than children born to non-smoking mothers. Significant interactions between maternal active smoking during pregnancy and child’s age were seen in those in the first and second quartiles of birthweight. Moreover, rapid growth in infancy was observed in the second quartile of birthweight.

**CONCLUSIONS:**

The effect of maternal active smoking during pregnancy on childhood growth was more apparent among children in the second quartile of birthweight.

## INTRODUCTION

In recent years, the ‘Developmental Origins of Health and Diseases’ (DOHaD) hypothesis, along with the established ‘foetal programming’ and ‘Barker’s hypothesis’, have been employed to explain the mechanisms of childhood body mass index (BMI, kg/m^2^) and development^1^. These hypotheses describe the association between a specific growth path, such as slow foetal growth and subsequent rapid infant growth, and the development of chronic diseases in adulthood^1–5^. Based on these findings, birthweight might be considered as an important factor influencing future morbidity and mortality.

Because birth weight is significantly associated with infant mortality^6^, stratified analyses by birthweight have examined the association between various perinatal factors, such as maternal active smoking during pregnancy and infant mortality. For instance, Hernández-Diaz et al.^7^ described infant mortality among smoking versus non-smoking mothers. Although infant mortality increased as birthweight decreased among infants in both groups, a ‘birthweight paradox’ was reported^7^, which suggested that the birthweight-specific mortality rate curve of infants born to smoking mothers crossed over that of infants born to non-smoking mothers at approximately 2000–2250 g. The authors explained this paradox by using directed acyclic graphs (DAGs) to consider causal models for the effects of unmeasured factors on birth weight and infant mortality^7^. Thus, it might be necessary to consider potential effects on birthweight from perinatal exposure to certain factors, such as maternal smoking, which impact on childhood health outcomes.

It has been suggested that maternal active smoking during pregnancy is a major factor affecting foetal and childhood BMI^8–17^. We have previously described this association using data from national (Japan Environmental and Children’s Study, JECS) and community-based birth cohort studies in Japan (Project Koshu)^18–24^. We found that there were both direct and indirect correlations between maternal active smoking during pregnancy and rapid infant growth^24^. We speculated that breastfeeding and birthweight might be intermediate factors between maternal active smoking during pregnancy and rapid infant growth^24^. Furthermore, because reports suggest that rapid growth during infancy could be associated with subsequent obesity^25–27^, describing the trajectory of childhood BMI might be important in determining their future weight status.

Therefore, in addition to maternal active smoking, there might be other potential factors during pregnancy that are associated with birthweight and childhood BMI. Since these potential factors, which were impossible to measure, might be independent from maternal active smoking during pregnancy, the effect of the latter on childhood BMI might vary by birthweight. It is, therefore, necessary to describe the trajectory of childhood BMI stratified by birthweight.

It is also necessary to conduct individual growth analysis, including both the individual and age as different-level variables. Therefore, this multilevel analysis was appropriate for longitudinal data, which were obtained repeatedly at different time points.

Thus, this study aimed to examine differences in the effect of maternal active smoking during pregnancy on childhood growth based on trajectories of childhood BMI z-scores, using multilevel model analyses stratified by birthweight quartiles.

## METHODS

### Study design and participants

This study is part of Project Koshu (formerly Project Enzan), a dynamic, ongoing Japanese, community-based, prospective cohort study that began in 1988. Details of this project have been described previously^18–24^. Participants were mothers who completed questionnaires during their pregnancy registration and whose infants were born between 1 April 1991 and 31 March 2003, in Koshu City, Japan. Koshu City has a population of 31000, with approximately 150 births each year. Because the birthweight distribution significantly differs between multiple and singleton pregnancies, women with multiple pregnancies were excluded.

This study was approved by the ethical review board of the University of Yamanashi School of Medicine and was conducted in accordance with the Guidelines Concerning Epidemiological Research (Ministry of Education, Culture, Sports, Science, and Technology and Ministry of Health, Labour and Welfare, Japan), with cooperation from the Koshu City administration office. Participants provided written informed consent.

### Data collection

Smoking status of pregnant women were recorded using a questionnaire during pregnancy registration. In Japan, maternal/child health laws require expectant mothers to register their pregnancies to access healthcare services. In the study area, almost all expectant mothers were registered by 18 weeks of gestation. Smoking status was categorised dichotomously; the ‘smoking mother’ category only included those participants who answered ‘smoking’, and the ‘non-smoking mother’ category included those who answered ‘have quit smoking’ or ‘have never smoked’ when asked about their smoking status.

Data regarding birth length and weight, birth order, and gestational week at delivery were obtained from the Maternal and Child Health Handbook, which is an official publication containing guidelines for obstetric professionals and pregnant women. Additionally, pregnant women used a booklet to record their health status during pregnancy. Childhood height and bodyweight data were collected via physical measurements taken during medical check-ups conducted when the children were 3 and 5 years old and when the children attended grades 2 and 4 of elementary school (i.e. ages 7–8 and 9–10 years, respectively). Height was measured using a stadiometer (unit: 0.1 cm), and bodyweight was measured using conventional weighing scales (unit: 100 g). BMI z-scores of children aged 0, 3, 5, 7–8, and 9–10 years, which were based on WHO standards, were used to adjust the differences in BMI for each month of age within the same age group^28^.

### Statistical analysis

Based on birth order and sex, children were initially categorised into quartiles of birthweight. The individual growth analysis method (SAS PROC MIXED) was used to compare childhood BMI z-score trajectories between children born to smoking and non-smoking mothers in each quartile. We did not exclude participants for whom BMI z-score data were missing, as SAS PROC MIXED automatically handles missing data using maximum likelihood. This automatic imputation was a particular strength of this method compared with other statistical methods such as the generalised estimating equation. As our previous findings indicated nonlinearity in the slopes for BMI and BMI z-scores, adopting the approach used by Fitzmaurice et al.^29^, we used the following model to explore the differences in the slopes for each interval between the ages of measurement^23^:

BMI z-score*_it_* = *β_1_* + *β_2_*×Age_it_ + *β_3_*×Maternal smoking status*_i_* + *β_4_*×Age*_it_*×Maternal smoking status*_i_* + *β_5_*×Maternal BMI before pregnancy*_i_* + e*_it_* where *i* represents the individual, *t* represents time, *β_1-5_* represent parameters, and *e* is the error term. In the final models, years were used to form dummy variables for time. Sample clustering within individuals was addressed. In this analysis, individual BMI data recorded at birth and at least once after the children were 3 years old were used. Individual BMI z-scores, which were based on WHO standards, were used to adjust differences in BMI for each month of age within age groups^28^. We calculated BMI z-scores for each group at each age using the solution from the final model to describe the trajectories. For example, BMI z-scores for boys aged 3 years were calculated as follows:

BMI z-score at 3 years of age = intercept (*β_1_*) + *β_2_* at 3 years of age + *β_3_* of Smoking (0 or 1) + *β_4_* at 3 years of age and Smoking (0 or 1) + *β_5_* ×Maternal BMI before pregnancy (kg/m^2^).

All analyses were conducted using SAS version 9.4 (SAS Institute, Inc., Cary, NC, USA) statistical software.

## RESULTS

The study participants were 1955 women and their single-born infants, for whom complete data for birthweight, maternal pre-pregnancy BMI, and smoking status during pregnancy were available. Birthweight and anthropometric data were collected from 1950 (at birth, 99.7%), 1643 (at age 3 years, 84.0%), 1517 (at age 5 years, 77.6%), 1487 (at age 7–8 years, 76.1%), and 1491 (at age 9–10 years, 76.3%) children. Children were stratified by quartile of birthweight based on sex and birth order. The approximate range of median, 25th percentile and 75th percentile of birthweight in each group was 3000–3100 g, 2700–2900 g, and 3200–3400 g, respectively (Table 1).

**Table 1:** Quartile of birthweight (g) stratified by sex of children and parity, Project Koshu, Japan, 1991–2003 (N=1955)

*Sex and parity*	*Quartile 1*	*Quartile 2*	*Quartile 3*	*Quartile 4*
Male and first children	<2794	2794–3004	3004–3240	≥3240
Female and first children	<2764	2764–2997	2997–3229	≥3229
Male and second or more children	<2860	2860–3105	3105–3382	≥3382
Female and second or more children	<2802	2802–3050	3050–3310	≥3310

There were 1015 (51.9%) boys and 826 (42.3%) first born children. In total, 128 (6.6%) mothers smoked during pregnancy. The mean maternal age was 28.9 years (SD: 4.3). The mean maternal pre-pregnancy BMI was 20.7 kg/m^2^ (SD: 2.8), and the mean birthweight of the infants was 3061 g (SD: 392.7). The smoking rate was highest in the first quartile (9.5%). The pre-pregnancy maternal BMI was the highest in the fourth quartile, and gestational age at delivery was the longest (Table 2).

**Table 2:** Characteristics of participants in each quartile, Project Koshu, Japan, 1991–2003 (N=1955)

*Characteristics*	*Quartile 1 (n=487)*	*Quartile 2 (n=485)*	*Quartile 3 (n=488)*	*Quartile 4 (n=495)*
Male children	252 (51.8)	253 (52.2)	253 (51.8)	257 (51.9)
First children	206 (42.3)	205 (42.3)	206 (42.2)	209 (42.2)
Smoking mother	46 (9.5)	38 (7.8)	25 (5.1)	19 (3.8)
Maternal age (years)	28.8±4.2	28.7±4.2	28.7±4.2	29.4±4.7
Maternal BMI before pregnancy	20.5±3.0	20.5±2.7	20.6±2.7	21.4±2.9
Birth order	1.8±0.8	1.8±0.8	1.8±0.8	1.9±0.8
Gestational weeks at delivery	38.0±1.6	39.0±1.2	39.3±1.1	39.6±1.1
Birthweight (g)	2579.5±234.5	2938.7±79.1	3169.4±95.6	3546.3±223.3
BMI at different ages				
At birth	11.7±0.9	12.4±0.8	13.0±0.8	13.8±1.0
3 years	15.4±1.3	15.8±1.3	15.9±1.2	16.0±1.3
5	15.3±1.6	15.5±1.6	15.6±1.4	15.8±1.5
7–8	15.9±2.3	16.0±2.3	16.1±1.9	16.3±2.2
9–10	17.0±3.1	17.2±3.1	17.1±3.1	17.4±3.1

Data given as n (%) or mean±standard deviation (SD). BMI: body mass index (kg/m^2^).

Multilevel analyses showed that in the first quartile, maternal active smoking during pregnancy had a significant association with childhood BMI (p=0.007). In the same quartile, the interaction between children’s age and maternal active smoking during pregnancy was significant (F=0.02) (Table 3). In the second quartile, maternal active smoking during pregnancy had a marginally significant association with childhood BMI (p=0.099). In the same quartile, the interaction between children’s age and maternal active smoking during pregnancy was significant (F=0.002) (Table 3).

**Table 3:** Fixed effects about body mass index (BMI) z-score in each age of children, smoking status of their mother, and interaction between each age and smoking status of their mother, Project Koshu, Japan, 1991–2003 (N=1955)

*Factor*	*Quartile*		*t*	*p*	*Quartile*		*t*	*p*
	
	*E*	*SE*			*E*	*SE*		
**Intercept**	−2.61	0.25	−10.59	<0.0001	−1.46	0.23	−6.34	<0.0001
**Childhood age**			(F<0.0001)				(F<0.0001)	
3 years	1.66	0.22	7.73	<0.0001	1.58	0.20	8.09	<0.0001
5	2.03	0.22	9.37	<0.0001	1.67	0.21	8.07	<0.0001
7–8	1.94	0.25	7.87	<0.0001	1.50	0.23	6.49	<0.0001
9–10	2.24	0.25	8.87	<0.0001	1.42	0.25	5.64	<0.0001
**Maternal smoking during pregnancy**	0.37	0.14	2.72	0.007	0.19	0.11	1.65	0.10
Childhood age*			(F=0.02)				(F=0.002)	
3 years	−0.33	0.22	−1.48	0.14	−0.69	0.20	−3.39	0.0008
5	−0.64	0.23	−2.82	0.005	−0.80	0.22	−3.74	0.0002
7–8	−0.48	0.26	−1.86	0.06	−0.65	0.24	−2.71	0.007
9–10	−0.68	0.26	−2.56	0.01	−0.44	0.26	−1.68	0.09
**Maternal BMI before pregnancy**	0.038	0.0102	3.69	0.0002	0.0243	0.0095	2.57	0.01
**Intercept**	−0.91	0.24	−3.77	0.0002	−0.93	0.25	−3.71	0.0002
**Childhood age**			(F<0.0001)				(F=0.83)	
3 years	0.67	0.22	3	0.003	0.33	0.30	1.13	0.26
5	0.81	0.23	3.5	0.0005	0.41	0.31	1.35	0.18
7–8	0.82	0.26	3.17	0.0016	0.50	0.37	1.33	0.18
9–10	0.86	0.27	3.24	0.001	0.35	0.40	0.87	0.39
**Maternal smoking during pregnancy**	0.10	0.14	0.71	0.48	0.108	0.17	0.64	0.52
Childhood age*			(F=0.44)				(F=0.61)	
3 years	−0.11	0.23	−0.47	0.64	−0.39	0.30	−1.29	0.20
5	−0.35	0.24	−1.47	0.14	−0.46	0.31	−1.48	0.14
7–8	−0.36	0.27	−1.36	0.17	−0.59	0.38	−1.56	0.12
9–10	−0.34	0.27	−1.23	0.22	−0.41	0.41	−1.00	0.32
**Maternal BMI before pregnancy**	0.023	0.0095	2.43	0.02	0.055	0.0094	5.84	<0.0001

BMI: body mass index (kg/m^2^).

* Children of mothers smoking during pregnancy. E: estimate. SE: standard error.

**Figure 1:**
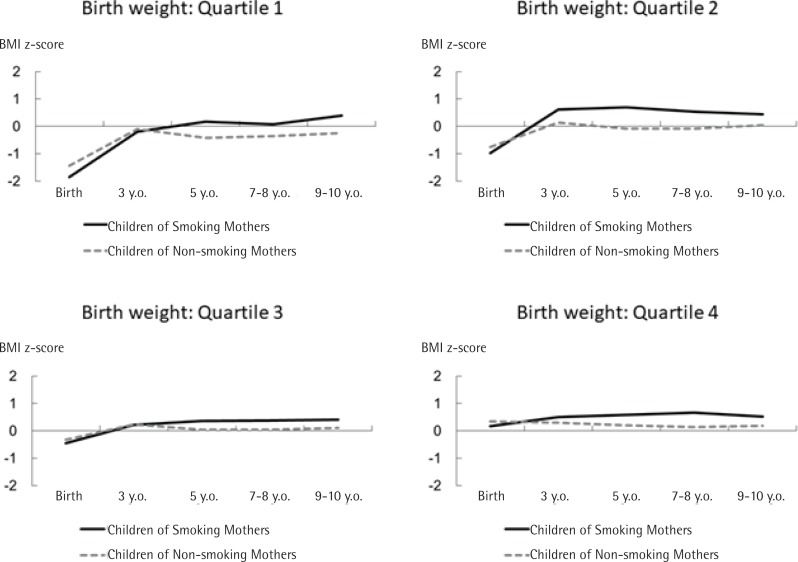
Body mass index (BMI) z-score trajectories of children between smoking and non-smoking mothers in each quartile of birthweight calculated by individual growth analysis, Project Koshu, Japan, 1991–2003 (N=1955)

In every quartile, although children born to smoking mothers were leaner at birth, their BMI z-scores increased rapidly by the age of 3 years (the 2nd and 4th quartiles); after the age of 3 years (the 1st and 3rd quartiles), these children were larger than those born to non-smoking mothers. The difference in birthweight trajectory between children born to smoking and non-smoking mothers was larger in the first and second quartiles than in the other groups (Figure 1).

## DISCUSSION

The present study examined differences in the effect of maternal active smoking during pregnancy on childhood growth based on trajectories of childhood BMI z-scores using multilevel model analyses stratified by quartile of birthweight. Regardless of sex and parity, the largest difference in the effect of maternal smoking on childhood BMI was observed when the birthweight was lower than the median value. In addition, rapid growth during infancy, which could be associated with subsequent obesity^25–27^, was observed in the second quartile of birthweight. Therefore, in the second quartile of birthweight, maternal active smoking during pregnancy might contribute to an increase in childhood BMI.

Many factors, including maternal active smoking during pregnancy, sex of the child, parity, gestational age, maternal age, pregestational maternal weight, gestational weight gain, hypertensive disorders during pregnancy, and gestational diabetes, have been associated with the birthweight of the infant^30–36^. Thus, to examine the association between maternal active smoking during pregnancy and birthweight, it is necessary to consider these factors as confounders or effect modifiers. Since the subjects were stratified based on sex and parity within the birthweight quartiles in this study, the effect of these factors might be limited. In addition, because Japanese vital statistics showed that maternal age might be associated with parity^37^, the effect of maternal age on birthweight might be partially controlled. However, the effect of other factors, including potential confounding factors, was not considered in the present study and might have impacted our results. Since Hernández-Diaz et al.^7^ evaluated the effect of these potential factors on birthweight and infant mortality using DAGs, our results could be interpreted by similar methods.

Our previous study on the effect of maternal active smoking during pregnancy on birthweight, which adjusted for clinical, socioeconomic, and maternal lifestyle factors, showed that maternal active smoking during pregnancy might reduce the birthweight by 120–150 g and approximately 130 g in this study area and nationwide, respectively^19,20^. Therefore, similar to the findings in the report by Hernandez-Diaz et al.^7^, which demonstrated that maternal active smoking during pregnancy shifted the distribution of infant mortality, smoking also shifts the distribution of birthweight to a lower weight. However, since the birthweight in the first quartile and mean birthweight in Japan were <2800 g and approximately 3000 g, respectively, factors other than maternal active smoking during pregnancy might have reduced the birthweight in the first-quartile group.

In addition to birthweight, factors like short gestational age also reduced the BMI in early childhood^38–40^. Infants in the first quartile might have a reduced birthweight due to maternal active smoking during pregnancy and other factors like gestational age. In view of these findings, the impact of maternal active smoking during pregnancy on childhood BMI might be underestimated in infants with relatively low birthweight.

In contrast, birthweights in the third and fourth quartiles were higher than the median regardless of maternal active smoking during pregnancy, implying the effect of opposing factors that increase birthweight, like appropriate gestational weight gain. The concept of DOHaD suggests that mismatched foetal and early infant environments might adversely affect future health status^41^. Therefore, in these quartiles, appropriate foetal factors that increase birthweight might reduce the adverse effects of maternal active smoking during pregnancy on childhood BMI.

Our results suggest that when determining the effect of maternal active smoking on foetal and childhood BMI, it is necessary to consider factors other than maternal active smoking during pregnancy as both confounding and effect modification factors.

### Strengths and limitations

This study has certain limitations. First, as it was conducted in only one rural Japanese area, the results might not be generalizable. However, in previous studies, the effect of maternal active smoking during pregnancy on birthweight in this area was similar to that observed nationwide. Additionally, the validity of the maternal smoking status might have been low as this information was obtained using a questionnaire. However, the effect of differential misclassification, which occurs when participants are unwilling to admit smoking, might be small. Finally, the period effects due to the long study period could have influenced the results. This study also has several strengths. First, although the follow-up period was relatively long, the follow-up rate was almost 80% for each age group. Thus, selection bias might be minimal. In addition, because the number of participants was large, the statistical power might be relatively high, although stratified analyses were conducted.

## CONCLUSIONS

The effect of maternal active smoking during pregnancy on childhood growth was more apparent among children in the second quartile of birthweight; rapid growth in infancy was also observed in children of smoking mothers in this group. It is necessary to consider other potential factors that modify the effect of maternal smoking during pregnancy on childhood BMI.
